# Polychondrite chronique atrophiante

**DOI:** 10.11604/pamj.2015.20.34.5898

**Published:** 2015-01-14

**Authors:** Madiha Mahfoudhi, Sami Turki

**Affiliations:** 1 Service de Médecine Interne A, Hôpital Charles Nicolle, Tunis, Tunisie

**Keywords:** Polychondrite atrophiante, vascularite, calcifications, trachée, atrophic polychondritis, vasculitis, calcifications, trachea

## Image en médecine

La polychondrite chronique atrophiante est une inflammation des cartilages. C'est unemaladie rare, qui se caractérise par des manifestations viscérales polymorphes. Patient âgé de 54 ans hospitalisé pour une douleur et une cyanose du pavillon de l'oreille droite. Il avait une aphtose buccale, une voix rauque, une douleur à la pression du cartilage nasal, une arthrite touchant les interphalangiennes proximales et distales ainsi que des lésions purpuriques infiltrées au niveau de la jambe avec à la biopsie un aspect de vascularite leucocytoclasique. L'examen ORL a trouvé une hypoacousie. Les examens ophtalmologiques et neurologiques étaient normaux. A la biologie, il avait une anémie normochrome normocytaire (Hb: 11 g/dl) associée à une accélération de la vitesse de sédimentation. Les bilans rénaux et hépatiques étaient sans anomalies. Le bilan immunologique était négatif, ainsi que le bilan de thrombophilie (protéine Cet S et anti-thrombine) éliminant une vascularite ou une connectivite. La TDM laryngo-trachéale a objectivé des calcifications tragiennes, des anneaux trachéaux et de la partie initiale des bronches souches. La biopsie du pavillon a confirmé des lésions de chondrite et périchondrite. La ponction sternale et la biopsie ostéo-médullaire ont permis d’éliminer une hémopathie ou une myélodysplasie. Le diagnostic d'une polychondrite atrophiante compliquée d'une vascularite cutanée a été retenu. Le malade a été traité par corticothérapie (3 boli de solumédrol et relais par prednisone 1mg/kg/j) avec diminution progressive des doses. L’évolution était marquée par la reprise d'un aspect normal du pavillon et la disparition des anomalies cliniques et biologiques.

**Figure 1:**
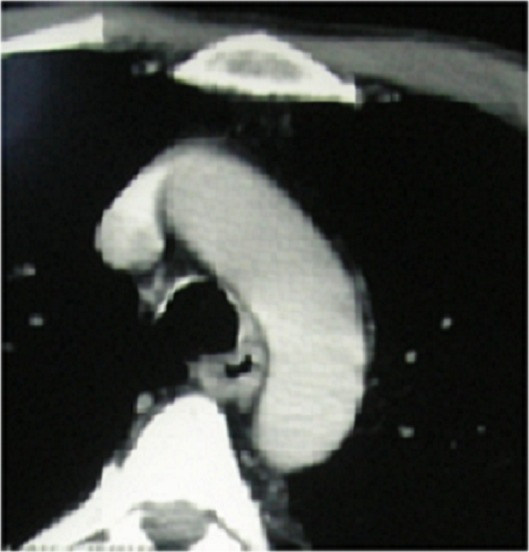
TDM laryngo-trachéale:calcifications des anneaux trachéaux

